# Patient and Public Involvement (PPI) in preclinical research: A scoping review protocol

**DOI:** 10.12688/hrbopenres.13303.2

**Published:** 2021-08-31

**Authors:** Pádraig Carroll, Adrian Dervan, Anthony Maher, Ciarán McCarthy, Ian Woods, Rachel Kavanagh, Cliff Beirne, Geoff Harte, Dónal O'Flynn, Paul Murphy, John Quinlan, Alice Holton, Sarah Casey, Frank Moriarty, Éimear Smith, Fergal J. O'Brien, Michelle Flood

**Affiliations:** 1 School of Pharmacy and Biomolecular Sciences, Royal College of Surgeons in Ireland, University of Medicine and Health Sciences, Dublin, D02 YN77, Ireland; 2 Tissue Engineering Research Group (TERG), Department of Anatomy and Regenerative Medicine, Royal College of Surgeons in Ireland, University of Medicine and Health Sciences, Dublin, D02 YN77, Ireland; 3 Advanced Materials and Bioengineering Research (AMBER) Centre, Trinity College Dublin, D02 W085 & RCSI Dublin, Dublin, D02 YN77, Ireland; 4 c/o Irish Rugby Football Union (IRFU) Charitable Trust, Dublin, D04 F720, Ireland; 5 Faculty of Sports and Exercise Medicine, (RCPI & RCSI), RCSI House, 121 St Stephen’s Green, Dublin 2, D02 H903, Ireland; 6 RCSI Library, Royal College of Surgeons in Ireland, University of Medicine and Health Sciences, Dublin, D02 P796, Ireland; 7 Tallaght University Hospital, Tallaght, Dublin, D24 NR04, Ireland; 8 National Rehabilitation Hospital, Dún Laoghaire, Dublin, Ireland

**Keywords:** Patient and public involvement, patient and public engagement, consumer involvement, public involvement in research, preclinical research

## Abstract

**Introduction: **Patient and public involvement (PPI) aims to improve the quality, relevance, and appropriateness of research and ensure that it meets the needs and expectations of those affected by particular conditions to the greatest possible degree. The evidence base for the positive impact of PPI on clinical research continues to grow, but the role of PPI in preclinical research (an umbrella term encompassing ‘basic’, ‘fundamental’, ‘translational’ or ‘lab-based’ research) remains limited. As funding bodies and policymakers continue to increase emphasis on the relevance of PPI to preclinical research, it is timely to map the PPI literature to support preclinical researchers involving the public, patients, or other service users in their research. Therefore, the aim of this scoping review is to explore the literature on patient and public involvement in preclinical research from any discipline.

**Methods:** This scoping review will search the literature in Medline (PubMed), Embase, CINAHL, PsycINFO, Web of Science Core Collection, Scopus, and OpenGrey.net to explore the application of PPI in preclinical research. This review will follow the Joanna Briggs Institute (JBI) guidelines for scoping reviews. It will be reported according to the Preferred Reporting Items for Systematic reviews and Meta-Analyses extension for Scoping Reviews (PRISMA-ScR). Two reviewers will independently review articles for inclusion in the final review. Data extraction will be guided by the research questions. The PPI advisory panel will then collaboratively identify themes in the extracted data.

**Discussion: **This scoping review will provide a map of current evidence surrounding preclinical PPI, and identify the body of literature on this topic, which has not been comprehensively reviewed to date. Findings will inform ongoing work of the research team, support the work of other preclinical researchers aiming to include PPI in their own research, and identify knowledge and practice gaps. Areas for future research will be identified.

## Introduction

Involving patients and the public in research is increasingly recognised as being important to help ensure that the research focuses on issues relevant to them. This approach, usually referred to as patient and public involvement or ‘PPI’ is most commonly defined as research conducted ‘with’ or ‘by’ members of the public rather than ‘to’, ‘about’ or ‘for’ them ^1^. PPI can involve patients at all stages of the research process, from identifying research priorities to disseminating findings ^2^, and it takes many forms. Examples include working with funders to prioritise research in certain areas, offering advice as members of a steering committee, collaborating on the development of research materials, and undertaking interviews with research participants ^1^. Two main arguments underpin the role of PPI in research, namely, the moral/ethical and methodological perspectives. The moral/ethical argument highlights that people affected by research should have a say in how it is conducted, and that as research is primarily funded through their taxes, the public have a right to input. Methodologically, PPI may enhance how research is conceptualised, designed and conducted, thus strengthening impact ^3– 5^. Finally, a third rationale for PPI has been identified, describing the ‘process’ related values of PPI. These values are associated with ‘doing’ PPI and include equality, respect and openness between researchers and PPI partners ^6^.

Much of the rise in PPI activity in recent years can be attributed to policymaker’s and funding bodies’ strategic commitment to PPI and the development of specific infrastructure to support its implementation. Organisations including National Institute for Health Research (NIHR) INVOLVE ^7^ in the UK and the Health Research Board (HRB) ^8^ in Ireland have been central to promoting, funding, and developing PPI ^9^. In Ireland, initiatives such as funding the development of the National PPI Network ^10^ and the launch of the HRB Open Research Public and Patient Involvement collection has provided a platform to promote PPI and enable researchers to collaborate and share best practices ^11^. Furthermore, through necessitating the inclusion of a PPI strategy as part of grant applications, funding bodies have ensured its establishment in research practice ^12^. In parallel, the evidence base to support the role of PPI in health and social care research has grown over the past decade. Several formal reviews have provided evidence of PPI’s positive impacts ^13, 14^. Some of the benefits identified include enhanced quality and appropriateness of research ^14^, empowerment of service users ^13^, and increased awareness of the patient’s lived experience for researchers ^15^. While many benefits are evident, it is important to note that inconsistencies in designing and reporting PPI have limited the ability to synthesise these findings and establish their implications ^15, 16^. Recent advancements such as the standardisation of reporting guidelines should support improvements in reporting of PPI in future research ^17^.

While significant progress has been made with the development of an evidence base and implementation of good practice guidelines for PPI in clinical research, the implementation of PPI in preclinical research (an umbrella term used to describe ‘basic’, ‘laboratory’, ‘fundamental’, or ‘translational’ research) is comparatively less well established ^18^. In many ways this is not surprising, as the roots of PPI lie within the health and social care sector ^19^. However, there is evidence that preclinical researchers aiming to realise the benefits of PPI and/or respond to funders’ requirements have more recently begun to include PPI in their research ^18, 20, 21^. There are early indications that the incorporation of PPI in preclinical research is not without challenge. These obstacles primarily relate to (i) issues in communicating the nature and relevance of the proposed research, which may have no immediately identifiable clinical relevance, (ii) lack of established methods/approaches for PPI in preclinical research, and (iii) perceptions amongst preclinical researchers that PPI may not be useful ^22, 23^. Notwithstanding these challenges, it is widely accepted that PPI has an important role to play in preclinical research and efforts to develop it further need to be increased ^23^.

As part of a collaboration between the Royal College of Surgeons in Ireland (RCSI) University of Medicine and Health Sciences, the Irish Rugby Football Union (IRFU) Charitable Trust, and the Science Foundation Ireland (SFI) Advanced Materials and Bioengineering Research Centre (AMBER), a research partnership was created to develop an advanced scaffold-based biomaterial platform for spinal cord repair. The project team considered the involvement of seriously injured players in the research process to be an important opportunity to maximise the potential impact of this research. To realise this, a PPI Advisory Panel, comprising seriously injured rugby players, clinicians, and researchers, was formed to both oversee project progress and develop a PPI strategy for the project. The team initially aimed to identify and implement evidence-based PPI approaches to use as a basis for strategy development, but this was challenging due to the emergent nature of preclinical PPI. Examples of commonly used approaches in PPI for clinical research were examined, but panel members struggled to identify how they might be readily included in the context of the Spinal Cord Repair Project. The panel members felt that there was merit in formally reviewing available examples of preclinical PPI as a starting point, to help inform the spinal cord project and support other preclinical research teams experiencing similar challenges. 

A scoping review was identified as the most appropriate methodology to achieve this. To our knowledge, there are no scoping reviews of PPI in preclinical research in any preclinical scientific disciplines. We identified one systematic review by Evans *et al.* that focused on antimicrobial drug development research ^24^, and one project database review by Nunn *et al.* that focused on genomics ^25^. However, both reviews were deliberately narrow in focus to meet specific needs. The antimicrobial review found no papers for inclusion (i.e. an ‘empty’ review) and the genomics study reviewed the databases of 96 publicly available human genomics initiatives rather than wider published, peer reviewed literature. While both studies are important in their own disciplines, they did not aim to identify approaches that may be more widely applicable in preclinical research. Therefore, we designed a scoping review that would allow the comprehensive mapping of literature on PPI in preclinical research. This would support preclinical researchers incorporating this into their practice as well as highlighting knowledge gaps and informing future research opportunities. As well as contributing towards the evidence base for this emerging area, this scoping review will also have immediate impact on the development of our PPI strategy for the Spinal Cord Repair Project.

## Protocol

### Design

A scoping review was determined the most appropriate review to answer the research question. The framework will be based upon the Joanna Briggs Institute (JBI) guidelines for conducting scoping reviews ^26^, and reported according to the Preferred Reporting Items for Systematic Reviews Extension for Scoping Reviews (PRISMA-ScR) reporting guideline ^27 ^. The JBI guidelines build upon previous guidance in best practice for scoping review methodology ^28, 29^.

***Stage 1: Identifying the review question***

This review aims to identify and map the current literature on PPI in preclinical research. Findings will support preclinical researchers aiming to incorporate PPI in their research and will identify priority areas for future research. The following objectives were developed to guide the scoping review:

(1) To identify why researchers use PPI in preclinical research and what they aimed to achieve by including PPI in their work
(2) To map the volume and range of PPI approaches used by researchers in preclinical research
(3) To identify what benefits or challenges are reported by preclinical researchers including PPI in their research
(4) To explore the nature of impacts of PPI on preclinical research as reported by preclinical researchers

The Population, Concept and Context (PCC) mnemonic was used to develop the research question, as recommended by the JBI guidelines for scoping reviews ^26^. Through use of this technique, the following research question was developed: How do researchers incorporate PPI in preclinical research? Each PCC element is outlined below.

Population: For the purposes of this review, the population will refer to ‘preclinical research’, meaning ‘laboratory’, ‘basic’, ‘fundamental’ or ‘translational’ research. While most research is distinctly clinical or preclinical, some may contain elements of both. In the instance that a study considered for inclusion is not clearly clinical or preclinical, the following question will be posed: ‘Does this study have immediate clinical application?’ If no immediate clinical application is evident, the study will be deemed as preclinical and eligible for inclusion.

Concept: PPI as defined by NIHR ^1^. This includes PPI approaches used at any point in the research cycle that involves patients and/or the public. The terminology used in PPI continues to evolve and remains somewhat contested, and there are variations in how the terms are used internationally ^30^. Therefore, studies that use PPI principles but describe this using slightly different terms (e.g. community-based participatory research, co-production, or citizen science ^31^) will be eligible for inclusion; the search terms selected will reflect this as outlined in Table 2. According to the NIHR, ‘involvement’ does not refer to raising awareness of research, sharing knowledge or creating dialogue with the public. These activates, referred to as ‘engagement’ and ‘participation’, while having a degree of crossover, are distinct from involvement ^1^. At the initial search stage of the review, studies on engagement and participation will be included in order to ensure no studies are inadvertently overlooked due to variance in terminology. However, at the screening stage, the NIHR definition of involvement will be applied to exclude the studies which are about engagement or participation.

Context: Relevant publications from any country will be included. Studies in both academic and industry settings will be included.

***Inclusion and exclusion criteria***

Based on the review question and PCC, a set of inclusion criteria were developed. They are outlined in Table 1.

**Table 1.** Inclusion & exclusion criteria.

Inclusion criteria	Exclusion criteria
Preclinical research	Clinical research (i.e. research with potential for immediate clinical application)
Patient and public involvement (PPI)	Studies where the focus is on engagement or participation, rather than involvement
Reporting on PPI activities/initiatives that have been conducted in preclinical research	Discussion/commentary of the potential role of PPI in preclinical research
English language	Non-English language
Any location/any year	

***Stage 2: Identifying relevant studies***

In order to capture the literature on PPI in preclinical research as comprehensively as possible, no time or location restrictions will be applied. All study types will be included (quantitative, qualitative, mixed methods, descriptive studies, and grey literature) where they describe empirical work. Commentaries, discussions, policy, or opinion-based publications will not be included. Due to the time and costs associated with translation, this scoping review will be limited to studies in the English language.

***Search strategy***

The search strategy for this scoping review was developed in collaboration with a specialist librarian who carried out an initial search. Potentially eligible literature will be identified through searching the following databases: Medline (PubMed), PsycInfo, Embase, CINAHL, Web of Science Core Collection, Scopus, and OpenGrey.net. This range of database was chosen for inclusion due to the broad nature of the review question. Searches will combine PPI MeSH terms with preclinical research terms including ‘preclinical research’, ‘basic research’ and ‘translational research’. Due to the broad nature of preclinical science, all MeSH terms relating to basic research methods will be included i.e. ‘translational medical research’, ‘biomedical research’, ‘animal models’ and ‘drug development.’ A sample search strategy for the PubMed databases are provided in Table 2.

**Table 2.** PubMed database search strategy.

Search String No.	Population, Concept and Context Concept Search - #1 AND #2
**#1**	**Concept 1: Population: Preclinical research** **Key words:** (((“Biological Science Disciplines” [Mesh] OR “biological science*”) OR ("Translational Medical Research"[Mesh] OR “translational medicine” OR "translational medical")) OR ("Biomedical Research"[Mesh] OR “biomedical research”)) OR ("Animal Experimentation"[Mesh] OR “animal experiment*”)) OR ("Biological Assay"[Mesh] OR biological assay*”)) OR ("Drug Development"[Mesh] OR “drug development”)) OR ("Immunologic techniques"[Mesh] OR “immunologic techniques”)) OR ("Cytological techniques"[Mesh] OR “cytological techniques””)) OR (("Cells"[Mesh]) OR cells[Title/Abstract])) OR ("Device Approval"[Mesh] OR “device approval”)) OR ("Chemistry Techniques, Analytical” [Mesh] OR “analytical chemistry”)) OR (“In Vitro Techniques” [Mesh] OR (“in vitro “ AND techniques))) **MeSH:** “Biological Science Disciplines” [Mesh] OR "Translational Medical Research"[Mesh] OR "Biomedical Research"[Mesh] OR "Animal Experimentation"[Mesh] OR “Biological Assay"[Mesh] OR "Drug Development"[Mesh] OR "Immunologic techniques"[Mesh] OR "Cytological techniques"[Mesh] OR "Cells"[Mesh] OR "Device Approval"[Mesh] OR "Chemistry Techniques, Analytical” [Mesh] OR “In Vitro Techniques” [Mesh]
**#2**	**Concept 2: Concept: Patient and public involvement (PPI)** **Key words: **"Patient Participation"[Mesh] OR "patient participation" OR “patient participants” OR "Participatory Research" **MeSH:** "Patient Participation"[Mesh] OR “community based participatory research”[Mesh]

Further evidence sources will be identified from searching grey literature. Grey literature will be searched using a title-only search of OpenGrey.net and limiting searches to the first 100 results. This is likely to be sufficient to capture relevant research ^32^. Key journals will be hand searched for PPI texts including Health Expectations, Research Involvement and Engagement, and HRB PPI Collection. Finally, citation searching of the eligible studies will be conducted using Google Scholar to identify studies, followed by hand searching of included studies’ reference lists.

***Stage 3: Study selection***

Results of database searches will be converted into .enw format and transferred into Endnote X9. Studies found using Google Scholar will be directly exported to Endnote. Duplicates will be removed using Endnotes ‘find duplicates’ function. The final de-duplicated library will then be used for study selection.

This review will be carried out in accordance with the JBI Framework for conducting a scoping review ^26^, which builds upon the guidelines of Arksey and O’Malley and Levac ^28, 29^. These principles will be followed during the study selection process, starting with a review of titles and abstracts using the inclusion and exclusion criteria, followed by full text retrieval of potentially eligible studies to be further examined for eligibility. In advance of commencing the full screening process, each reviewer will independently screen ten included studies before comparing results to ensure consistency. Two researchers using Endnote will screen the title and abstract of each record independently before meeting to compare results Any discrepancies will be resolved via discussion or the use of a third reviewer (FM) if consensus cannot be reached. Following title/abstract screening, full text screening will take place using the same iterative process. Each reviewer will conduct full text screening independently before meeting to discuss and agree on final included studies. If consensus cannot be reached, a third reviewer will be consulted ^26^.

***Stage 4: Data extraction***

To extract the data from the included studies, a data extraction form will be developed in a spreadsheet format in accordance with the JBI guidelines ^33^. The following elements will be extracted from the selected studies:

1. Author(s)
2. Year of publication
3. Title
4. Origin/country of origin (where the source was published or conducted)
5. Study aims/purpose
6. Population
7. Methodology/methods
8. Intervention type, comparator and details of these (e.g. duration of the intervention) (if applicable). Duration of the intervention (if applicable)
9. Outcomes and details of these (e.g. how measured) (if applicable)
10. Key findings
11. Gaps in existing research

The selected elements are based upon JBI recommendations for charting and extracting data. Two reviewers will pilot the data extraction form by will conducting full data extraction on five selected sources to measure agreement. Data extraction will be completed separately, in duplicate. Any discrepancies will be resolved via discussion until consensus or through inclusion of a third reviewer to act as an arbitrator. Once the accuracy and comprehensiveness of the tool has been established, we will proceed to full data extraction. As scoping reviews are an iterative process ^29^, it is expected that the data extraction form will be refined and modified throughout the charting process. Once extracted, all data will be compiled into a spreadsheet via Microsoft Excel 2016.

***Stage 5: Collating, summarising and reporting of results***

The PRISMA-ScR guidelines will be used to report the outcomes of the review ^27^. A PRISMA flow diagram will be produced to show the number of included studies, and reasons for full-text study exclusion ^27^. Extracted data relating to study characteristics will be tabulated, and a brief descriptive analysis of the included studies and the types of evidence sources available will be developed as per the JBI guidelines ^26^. Final data extraction forms will be made available along with the finished scoping review. The research objectives will be used to guide data extraction, and key themes will then be identified collaboratively by the PPI Advisory Panel (see Step 6 below). Finally, our findings will be discussed in relation to gaps in the literature and future implications. We expect the process of presenting our findings to be an iterative process, with adaptions and refinements included. It is anticipated that findings will be presented in graphical and tabular formats with consideration given to making the findings accessible to all members of the PPI Advisory Panel. The rationale for how findings are presented and changes adopted through involvement of the PPI Advisory Panel will be described fully in the final scoping review manuscript.

***Step 6: Consultation with stakeholders***

Consultation with stakeholders is generally considered to be an optional step ^28, 29^, but it forms an integral part of this scoping review and reflects our commitment to PPI in this project. The primary stakeholder groups (i.e. people affected by spinal cord injury, clinicians, and the preclinical scientists) co-designed the review question. Three PPI partners (CM, GH, and DO’F) are involved of the development of this review as co-authors. They identified the need for this review and the benefits that it could have for the entire project team. Furthermore, they will be involved in reviewing the extracted data for the final review. The extracted data will be presented at the next available Advisory Panel Meeting and the team will identify key themes both for the Spinal Cord Repair Project and more generally for PPI in preclinical research. These findings will inform the development of the project PPI strategy, the scoping review manuscript, and a report aiming to make the findings accessible to preclinical researchers and PPI contributors. PPI partners will be involved at the commissioning, design, conceptualisation and manuscript preparation stages of the final review, aligning with our ambition for a ‘partnership’ level of involvement ^34^.

## Discussion

Through this scoping review, we will comprehensively map the literature and support preclinical researchers incorporating PPI into their practice. By collating current practices for preclinical PPI, we will highlight gaps in the literature and provide opportunity for future research. The proposed approach has the potential to enhance the review by embedding PPI throughout. Involving key stakeholder groups in setting review objectives and identifying themes will improve the relevance of the review. This review addresses gaps in the current PPI literature and may highlight priority areas for preclinical PPI research. Furthermore, consistent limitations in the current literature may be identified i.e. lack of impact assessment. As well as contributing towards the wider evidence base, the findings of this scoping review will help inform the development of a PPI strategy for the larger Spinal Cord Repair Project.

## Study status

At the time of publication, database searches are currently underway as outlined in the methods section.

## Data availability

No data are associated with this article.
