# Supplementary material for: Patient and Public Involvement (PPI) in preclinical research: A scoping review protocol
Source: HRB Open Res. 2021 Aug 31;4:61. Originally published 2021 May 28. [Version 2] doi: 10.12688/hrbopenres.13303.2 (PMC8420886; doi:10.12688/hrbopenres.13303.2)
Supplement: Peer review history

# Peer review: Reviewer response for version 2

**DOI:** 10.21956/hrbopenres.14593.r30168

**Published:** 2021-09-03

**Authors:** Jon Salsberg, Meghan Gilfoyle

**Affiliations:** 1 Public and Patient Involvement Research Unit, School of Medicine and Health Research Institute (HRI), University of Limerick, Limerick, Ireland

Thank-you to the authors for thoroughly exploring the suggested changes. This is well reflected in this revised document.

Is the study design appropriate for the research question?

Partly

Is the rationale for, and objectives of, the study clearly described?

Yes

Are sufficient details of the methods provided to allow replication by others?

Partly

Are the datasets clearly presented in a useable and accessible format?

Not applicable

Reviewer Expertise:

Public and Patient Involvement in health research, Mixed-methods research

We confirm that we have read this submission and believe that we have an appropriate level of expertise to confirm that it is of an acceptable scientific standard.

# Peer review: Reviewer response for version 1

**DOI:** 10.21956/hrbopenres.14481.r29574

**Published:** 2021-07-07

**Authors:** Jon Salsberg, Meghan Gilfoyle

**Affiliations:** 1 Public and Patient Involvement Research Unit, School of Medicine and Health Research Institute (HRI), University of Limerick, Limerick, Ireland

**1. Is the rationale for, and objectives of, the study clearly described?**

 (Yes, No, Partly)

**Yes. **

- Great to see the incorporation of the advisory panel throughout.

- In the introduction, you discuss “Two main arguments underpin the role of PPI in research”. I would argue there is a third important element that should be discussed, which involves the process related values for doing PPI.
  See the review by Gradinger et al., 2015; “Values associated with public involvement in health and social care research: a narrative review”

**2. Is the study design appropriate for the research question?**

 (Yes, No, Partly)

**Partly**

- A small point would be that it is unnecessary to include the word ‘structured’ regarding the scoping review, as arguably, most should follow this systematic design.
- In the design, the rationale for excluding literature that discusses engagement vs. involvement is highlighted. While I appreciate the reasoning for this, what may be problematic, is that you are including terms, like CBPR, which would define engagement the way Ireland/UK defines involvement. Specifically, in locations, like North America, where ‘patient engagement’ is the pinnacle term, you would inherently be excluding a body of literature from these areas, which you are including in your search terms (which is important).
- Further, please consider including community-based participatory research as a MeSH heading, as to my knowledge, this is the terms referred to in MeSH heading look-up.
- Further, you mention you are excluding policy-related literature, but then go on to mention you will be handsearching relevant literature, including policy documents, this is unclear.

**3. Are sufficient details of the methods provided to allow replication by others?**

 (Yes, No, Partly)

**Partly**

- Further detail regarding the screening process would be useful. Specifically, it is unclear how discrepancies between the two reviewers will be dealt with at the title and abstract level of screening.
- Will the Advisory Panel be involved in this stage? If so, please highlight here.

**4. Are the datasets clearly presented in a useable and accessible format?**

 Not applicable.

Is the study design appropriate for the research question?

Partly

Is the rationale for, and objectives of, the study clearly described?

Yes

Are sufficient details of the methods provided to allow replication by others?

Partly

Are the datasets clearly presented in a useable and accessible format?

Not applicable

Reviewer Expertise:

Public and Patient Involvement in health research, Mixed-methods research

We confirm that we have read this submission and believe that we have an appropriate level of expertise to confirm that it is of an acceptable scientific standard, however we have significant reservations, as outlined above.

# Peer review: Reviewer response for version 1

**DOI:** 10.21956/hrbopenres.14481.r29573

**Published:** 2021-06-14

**Authors:** Éidín Ní Shé

**Affiliations:** 1 School of Population Health, University of New South Wales, Sydney, Australia

This is a welcome scoping review protocol looking at capturing the evidence of PPI in preclinical research. The authors define pre-clinical as including "‘basic’, ‘fundamental’, ‘translational’ or ‘lab-based research".

 The protocol is very clear, and I have some minor suggestions for the authors to consider:

1. How will the PPI advisory panel members be supported to be involved in identifying themes? Will they be included as authors in any outputs, and if so, how will this be enabled?
2. It would be welcome if the authors considered extracting the levels of involvement of PPI partners during this stage. what levels of support and capacity were provided to them and what adoptions researchers enabled to support involvement, and what lessons are there for funders/universities.

I look forward to seeing the outputs from this review.

Is the study design appropriate for the research question?

Yes

Is the rationale for, and objectives of, the study clearly described?

Yes

Are sufficient details of the methods provided to allow replication by others?

Yes

Are the datasets clearly presented in a useable and accessible format?

Not applicable

Reviewer Expertise:

Health Systems, co-design, PPI

I confirm that I have read this submission and believe that I have an appropriate level of expertise to confirm that it is of an acceptable scientific standard.
